# Angiotensin receptor-neprilysin inhibitor improves coronary collateral perfusion

**DOI:** 10.3389/fcvm.2022.981333

**Published:** 2023-02-03

**Authors:** Kangbo Li, Victoria Kratzmann, Mengjun Dai, Nora Gatzke, Petra Rocic, Peter Bramlage, Olaf Grisk, Lubomir T. Lubomirov, Meike Hoffmeister, Martin A. Lauxmann, Oliver Ritter, Eva Buschmann, Michael Bader, Anja Bondke Persson, Ivo Buschmann, Philipp Hillmeister

**Affiliations:** ^1^Department for Angiology, Center for Internal Medicine I, Deutsches Angiologie Zentrum Brandenburg - Berlin, University Clinic Brandenburg, Brandenburg Medical School Theodor Fontane, Brandenburg an der Havel, Germany; ^2^Charité – Universitätsmedizin Berlin, Corporate Member of Freie Universität Berlin and Humboldt-Universität zu Berlin, Berlin, Germany; ^3^Max Delbrück Center for Molecular Medicine in the Helmholtz Association, Berlin, Germany; ^4^Department of Physiology and Pharmacology, College of Osteopathic Medicine, Sam Houston State University, Huntsville, TX, United States; ^5^Institute for Pharmacology and Preventive Medicine, Cloppenburg, Germany; ^6^Institute of Physiology, Brandenburg Medical School Theodor Fontane, Neuruppin, Germany; ^7^Institute of Biochemistry, Brandenburg Medical School Theodor Fontane, Brandenburg an der Havel, Germany; ^8^Faculty of Health Sciences Brandenburg, Joint Faculty of the Brandenburg University of Technology Cottbus – Senftenberg, The Brandenburg Medical School Theodor Fontane, University of Potsdam, Brandenburg an der Havel, Germany; ^9^Department for Cardiology, Center for Internal Medicine I, University Clinic Brandenburg, Brandenburg Medical School Theodor Fontane, Brandenburg an der Havel, Germany; ^10^Department of Cardiology, University Clinic Graz, Graz, Austria; ^11^German Center for Cardiovascular Research, Partner Site Berlin, Berlin, Germany; ^12^Institute for Biology, University of Lübeck, Lübeck, Germany

**Keywords:** angiotensin receptor-neprilysin inhibitor, angiotensin-converting enzyme inhibitor, kallikrein-kinin system, heart failure, myocardial infarction, coronary collateral perfusion, arteriogenesis

## Abstract

**Background:**

We investigated the pleiotropic effects of an angiotensin receptor-neprilysin inhibitor (ARNi) on collateral-dependent myocardial perfusion in a rat model of coronary arteriogenesis, and performed comprehensive analyses to uncover the underlying molecular mechanisms.

**Methods:**

A rat model of coronary arteriogenesis was established by implanting an inflatable occluder on the left anterior descending coronary artery followed by a 7-day repetitive occlusion procedure (ROP). Coronary collateral perfusion was measured by using a myocardial particle infusion technique. The putative ARNi-induced pro-arteriogenic effects were further investigated and compared with an angiotensin-converting enzyme inhibitor (ACEi). Expression of the membrane receptors and key enzymes in the natriuretic peptide system (NPS), renin-angiotensin-aldosterone system (RAAS) and kallikrein-kinin system (KKS) were analyzed by quantitative polymerase chain reaction (qPCR) and immunoblot assay, respectively. Protein levels of pro-arteriogenic cytokines were measured by enzyme-linked immunosorbent assay, and mitochondrial DNA copy number was assessed by qPCR due to their roles in arteriogenesis. Furthermore, murine heart endothelial cells (MHEC5-T) were treated with a neprilysin inhibitor (NEPi) alone, or in combination with bradykinin receptor antagonists. MHEC5-T proliferation was analyzed by colorimetric assay.

**Results:**

The *in vivo* study showed that ARNis markedly improved coronary collateral perfusion, regulated the gene expression of KKS, and increased the concentrations of relevant pro-arteriogenic cytokines. The *in vitro* study demonstrated that NEPis significantly promoted MHEC5-T proliferation, which was diminished by bradykinin receptor antagonists.

**Conclusion:**

ARNis improve coronary collateral perfusion and exert pro-arteriogenic effects *via* the bradykinin receptor signaling pathway.

## Introduction

Heart failure (HF) is a major negative prognostic factor in patients after myocardial infarction (MI). Despite this, efforts to improve myocardial repair have not been translated into clinical therapies. Post-MI HF remains a leading cause of morbidity and mortality worldwide, and is marked by a sharply rising prevalence in the Western population over age of 75 years (1). Recently, an angiotensin receptor-neprilysin inhibitor (ARNi) was approved as a first-in-class drug for the treatment of HF in both Europe and the U.S., representing a new milestone in pharmaceutical treatment for HF (2). ARNi is a sodium supramolecular complex of a neprilysin inhibitor (NEPi) (i.e., Sacubitril) and an angiotensin receptor blocker (ARB) (i.e., Valsartan) at a 1:1 ratio. Neprilysin (NEP), also known as neutral endopeptidase, enzymatically degrades natriuretic peptides (NPs), which includes atrial natriuretic peptide (ANP), B-type natriuretic peptide (BNP), and C-type natriuretic peptide (CNP). The natriuretic peptide system (NPS) plays a key role in cardiovascular homeostasis by regulating a wide spectrum of physiological processes, such as natriuresis and vasodilation. Therefore, NEPis are used clinically to counteract the defects of NPS during the pathological process of HF (3). However, because NEP also depletes angiotensin I (Ang I) and angiotensin II (Ang II), the NEPi (Sacubitril) is combined with the ARB (Valsartan) to further reduce the Ang II-induced vasoconstriction (4). Hence, the pharmacological mechanisms of ARNis are based on augmentation of NPS and inhibition of renin-angiotensin-aldosterone system (RAAS), thereby countering the damage caused by sustained neurohormonal overactivation of RAAS and sympathetic nervous system (SNS) in chronic HF (5).

**FIGURE 1:**
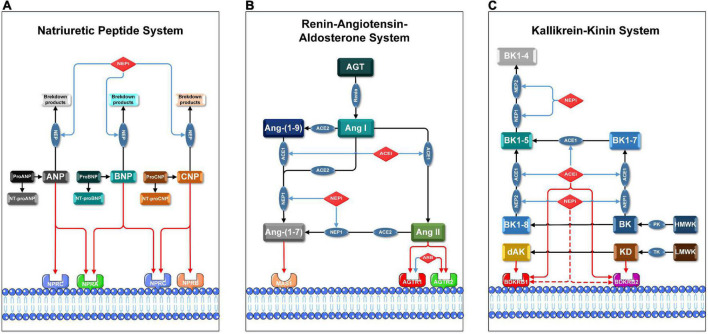
Pharmacological mechanisms of ARNis and ACEis in NPS, RAAS, and KKS. Blue lines indicate inhibitory or blocking effects, red lines indicate activating or stimulating effects, and red dashed lines indicate the hypothetical activating effects of NEPis on bradykinin receptors. AGT: angiotensinogen, Ang-(1-7): angiotensin-(1-7), Ang-(1-9): angiotensin-(1-9), BK1-4: bradykinin-(1-4), BK1-5: bradykinin-(1-5), BK1-7: bradykinin-(1-7), BK1-8: bradykinin-(1-8), dAK: des-Arg10-kallidin, HMWK: high-molecular-weight kininogen, LMWK: low-molecular-weight kininogen, NT-proANP: N-Terminal proANP, NT-proBNP: N-Terminal proBNP, NT-proCNP: N-Terminal proCNP, PK: plasma kallikrein, TK: tissue kallikrein. **(A)** Membrane receptors in NPS are NPRA, NPRB, and NPRC; key enzymes in NPS are NEP1 and NEP2. Precursor molecules of pro-ANP, pro-BNP, and pro-CNP break up into active ligands (ANP, BNP, and CNP) and corresponding inactive amino-terminal fragments (NT-proANP, NT-proBNP, and NT-proCNP), respectively. **(B)** Membrane receptors in RAAS are AGTR1, AGTR2, MAS1 proto-oncogene (MAS1); key enzymes in RAAS are ACE1 and ACE2. ACE1 converts Ang I to Ang II and subsequently activates AGTR1 and AGTR2. Either NEP1 or ACE2 converts Ang I or Ang II to Ang-(1-7), which then activates MAS1. **(C)** Membrane receptors in KKS are BDKRB1 and BDKRB2, both ACE1 and NEPs are involved in degradation of BK and KD in multiple steps. BK is cleaved by PK from HMWK, while KD is cleaved by TK from LMWK. BK and KD are the ligands of BDKRB2. BK and KD can be converted to BK1-8 and dAK, respectively. BK1-8 and dAK are the ligands of BDKRB1.

Yet, the cardiovascular hormone regulation modulated by ARNis is not limited to NPS and RAAS. Since NEP cleaves a wide range of peptides such as bradykinin (BK) and kallidin (KD), the kallikrein-kinin system (KKS) is also involved in the hormonal regulation by ARNi (Figure 1). KKS is a key proteolytic system regulating vascular permeability, blood pressure and collateral blood flow (6). KKS exerts its biological functions by stimulating two G protein-coupled receptors (GPCRs): bradykinin receptor 1 (BDKRB1) and bradykinin receptor 2 (BDKRB2). Notably, our group was the first to demonstrate that arteriogenesis is modulated by bradykinin receptor signaling (7). Here, arteriogenesis is defined as the remodeling and outgrowth of pre-existing collateral arteries following stenosis or occlusion. More specifically, arteriogenesis is an adaptive response that a small collateral arteriole (native collateral) converts into a larger conduit artery, thereby restoring the nutritive blood flow to ischemic area (8). Therefore, arteriogenesis is regarded as the most effective compensatory mechanism to prevent cardiovascular ischemia (9).

For decades, clinicians and scientists have focused on therapeutic arteriogenesis by investigating the pleiotropic roles of new or classical medications in this context (10). Indeed, our latest study verified that cerebral arteriogenesis can be therapeutically stimulated by an angiotensin-converting enzyme inhibitor (ACEi) through the bradykinin receptor signaling pathway (11). As the cornerstones in cardiovascular disease management, ACEis cause inhibition of angiotensin-converting enzyme (ACE, also known as kininase II). ACE not only converts Ang I to Ang II, but also degrades BK and bradykinin-(1-8) (BK1-8). Therefore, inhibition of ACE accumulates BK and BK1-8, which in turn activate the bradykinin receptors directly or indirectly (12). Interestingly, because NEP is a major kininase like ACE, it is speculated that NEPis can stabilize and activate bradykinin receptors like ACEis, thereby exerting further cardiovascular protective effects. Therefore, in our current study, we hypothesized that coronary arteriogenesis can be therapeutically enhanced beyond its natural time course by administration of ARNis, and the putative ARNi-induced pro-arteriogenic effects are based on activation of bradykinin receptors.

**FIGURE 2:**
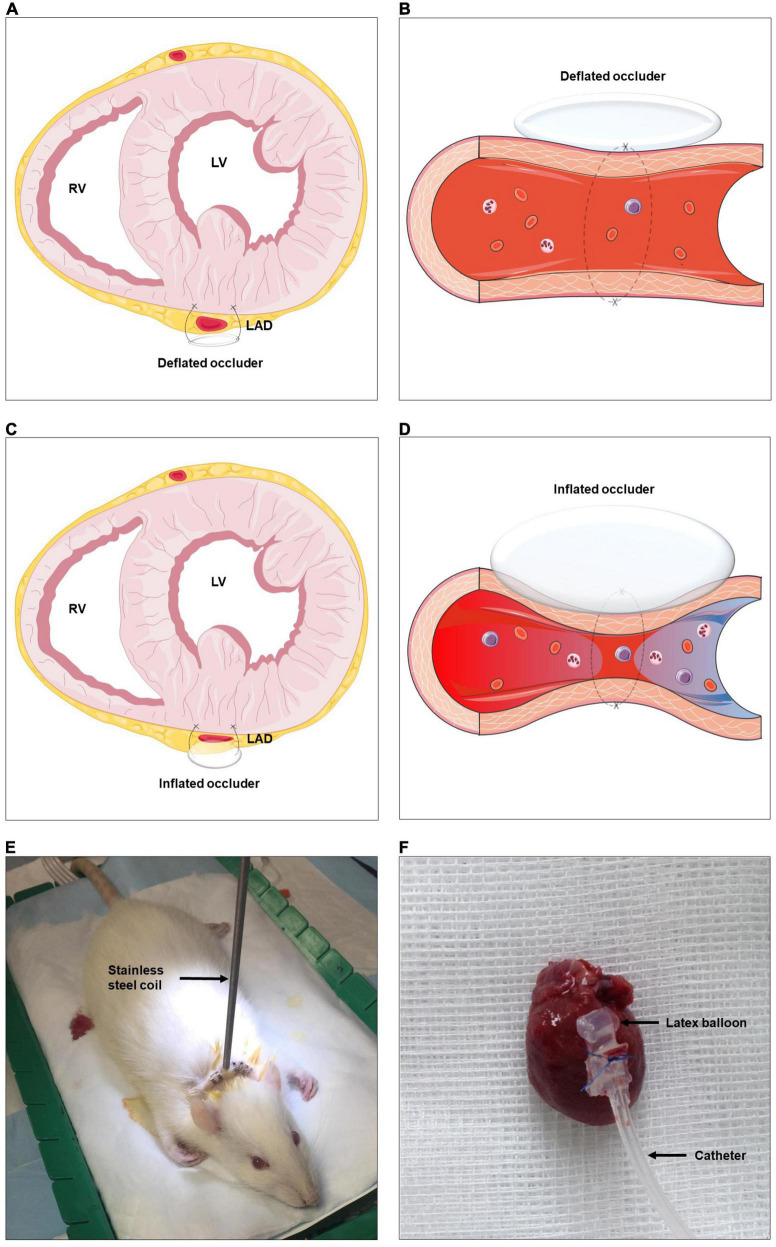
Schematic representation of rat model of coronary arteriogenesis. RV, right ventricle; LV, left ventricle; LAD, left anterior descending artery. **(A–D)** A highly flexible latex balloon catheter is fixed around the proximal LAD, compressed air is used to briefly and temporarily inflate the catheter, repetitively ligating the LAD, and thus stimulating coronary collateral development. **(E)** Established rat model with the stainless steel coil. **(F)** Sample of excised rat heart with the occluder. The figures were partly generated using Servier Medical Art, provided by Servier, licensed under a Creative Commons Attribution 3.0 unported license.

To investigate the role of ARNis on coronary collateralization, a suitable animal model is needed. By conducting a repetitive occlusion procedure on the left anterior descending artery (LAD), a rat model of coronary arteriogenesis has been established successfully (Figure 2) (13). The current study consists of three sub-projects. (1) First, we investigated the effects of ARNis on coronary arteriogenesis by assessing collateral-dependent myocardial perfusion in a rat model. Since the ARNi is a dual-acting complex composed of a NEPi and an ARB, the effects of the ARB (Valsartan) were also investigated. (2) Second, to uncover the underlying molecular mechanism of ARNi-induced coronary arteriogenesis, we analyzed mRNA and protein expression levels of the relevant membrane receptors and key enzymes, concentrations of pro-arteriogenic cytokines and mitochondrial DNA copy number. Since ACEi-induced cerebral arteriogenesis was characterized in our recent study (11), an ACEi (Ramipril) was also investigated for coronary arteriogenesis for comparison. (3) Finally, we investigated the role of the NEPi (Sacubitril) on murine endothelial cells (ECs) proliferation, and functionally validated whether the putative NEPi-induced pro-arteriogenic effects were modulated by the bradykinin signaling pathway *in vitro.*

## Materials and methods

### Surgical protocol

Male Sprague-Dawley rats (300–350 g) were sedated by using ketamine (75 mg/kg; i.p.) (Wirtschaftsgenossenschaft deutscher Tierärzte eG) and xylazine (5 mg/kg; i.p.) (Bayer AG) before endotracheal intubation. Endotracheal general anesthesia was maintained during surgery by using isoflurane (1.2% in 95% O_2_/5% CO_2_) (CP-Pharma Handelsgesellschaft mbH).

Left-sided thoracotomy was performed to expose the rat heart. The so called “occluder” consists of a latex balloon (a micro-pneumatic snare mounted within an umbrella sheath) and a catheter, the former part was implanted on the heart wall. In brief, a 5-0 Prolene suture was tied around the proximal LAD when the latex balloon was inflated with 0.6 ml of air. The latex balloon was connected to the catheter, which was protected by a stainless steel coil and externalized between the rat’s scapulae, and finally connected to an air pump machine outside of the animal cage. The machine was pumping according to a relevant procedure described hereinafter and supplying air through the catheter to the latex balloon. Consequently, the LAD was cyclically blocked and unblocked during the inflation and deflation of the latex balloon (Figure 2 and Supplementary Video).

### Repetitive occlusion procedure

It has been verified that the repetitive occlusion of a main coronary artery is an optimal method to establish animal models of coronary arteriogenesis (13). In addition, a moderate intensity and duration of myocardial ischemia are necessary to initiate collateral development (14). The repetitive occlusion procedure (ROP) began on the next day of occluder implantation surgery. The preset ROP consists of 7 × 24-h repetitive routines, and each 24-h individual routine again consists of 3 × 8-h repetitive routines, and each 8-h individual routine again consists of a 2-h and 20-min stimulating stage and followed by a 5-h and 40-min resting stage. Further, each stimulating stage again consists of 7 × 20-min repetitive routines, and each 20-min individual routine again consists of a 40-s balloon inflation and followed by an 18-min and 20-s balloon deflation.

### Microspheres–based myocardial perfusion measurement

The sensitivity and ability to measure myocardial perfusion in intact tissue using the stable isotope-labeled microspheres have been validated by comparison with the standard conventional radioactive method (15). Since the injected microspheres circulate in the blood and finally deposit in the region supplied by its corresponding artery, the deposited microsphere concentration is proportional to the blood flow. Given that the occluder was implanted around the LAD and the aim of our experiment was to evaluate coronary collateral perfusion, the LAD-dependent myocardial region distal to the occluder was referred to as the collateral dependent zone (CZ), and the left circumflex artery-dependent myocardial region was referred to as the normal zone (NZ). In order to ensure as many microspheres as possible enter the CZ, microspheres were injected rapidly during the occluder was inflated. In brief, 15 μl of 5 × 10^5^ isotope-labeled 15 μm-diameter microspheres were injected twice into the left ventricle (LV) on Day 0 (the day of occluder implantation) (Samarium STERIspheres, BioPAL, Inc.), and Day 7 (the day of animal sacrifice) (Gold STERIspheres, BioPAL, Inc). After sacrificing, 100 mg of NZ and CZ were collected, respectively and sent to BioPAL, Inc., for further analysis.

During the measurement of microspheres-based myocardial perfusion (MMP), the microsphere activity concentration was expressed as disintegrations per minute per gram (dpm/g). Here, MMP of different myocardial regions (CZ and NZ) at different time points (Day 0 and Day 7) were recorded, respectively. Specifically, MMP of CZ at Day 0, MMP of CZ at Day 7, MMP of NZ at Day 0 and MMP of NZ at Day 7 were represented by MMP*_*CZ(D*0)_*, MMP*_*CZ(D*7)_*, MMP*_*NZ(D*0)_*, and MMP*_*NZ(D*7)_*, respectively. MMP at Day 0 was regarded as a reference at baseline, and the difference value of MMP between Day 7 and Day 0 was represented by ΔMMP. Specifically, the difference value of MMP*_*CZ*_* between Day 7 and Day 0 was represented by ΔMMP*_*CZ*_* [MMP*_*CZ(D*7)_* – MMP*_*CZ(D*0)_*]; similarly, the difference value of MMP*_*NZ*_* between Day 7 and Day 0 was represented by ΔMMP*_*NZ*_* [MMP*_*NZ(D*7)_* – MMP*_*NZ(D*0)_*].

### Animal grouping and treatment protocol

All experimental animals were randomly assigned to the following groups (*n* = 6–8/group): (1) SHAM group: the occluder was implanted but without any procedures of inflation or deflation for 7 days, meanwhile, distilled water (0.5 ml per day) was administrated *via* gavage. (2) ROP-Ctrl group: ROP procedure was conducted for 7 days, meanwhile, distilled water (0.5 ml per day) was administrated *via* gavage. (3). ROP+ARB group: ROP procedure was conducted for 7 days, meanwhile, Valsartan (31 mg/kg per day) (Novartis International AG) was dissolved in 0.5 ml distilled water and administrated *via* gavage. (4). ROP+ACEi group: ROP procedure was conducted for 7 days, meanwhile, Ramipril (1 mg/kg per day) (AbZ-Pharma GmbH) was dissolved in 0.5 ml distilled water and administrated *via* gavage. (5) ROP+ARNi group: ROP procedure was conducted for 7 days, meanwhile, Sacubitril/Valsartan (68 mg/kg per day) (Novartis International AG) was dissolved in 0.5 ml distilled water and administrated *via* gavage.

### Analysis for mRNA expression of membrane receptors and key enzymes

Tissue samples from CZ (*n* = 6/group) were snap frozen and stored in liquid nitrogen before RNA isolation. Two cubic millimeter tissue from CZ was homogenized using the liquid nitrogen grinding method. Total RNA was extracted using the Trizol reagent (Thermo Fisher Scientific) in compliance with the manufacturer’s instructions. Quantitative analysis of RNA was performed using the Nanodrop™ Microvolume Spectrophotometer (Thermo Fisher Scientific). 1 μg of total RNA was reverse transcribed into cDNA by using the QuantiTect Reverse Transcription Kit (QIAGEN) and the peqSTAR thermal cycler (VWR International). The obtained cDNA was diluted in 60 μl RNAse/DNAase-free water. The quantitative polymerase chain reaction (qPCR) based analysis was performed by using the LightCycler^®^ 96 Real-Time PCR System (Roche). Each reaction system consists of 1 μl cDNA, 1 μl of forward/reverse primer each, 7 μl RNase/DNase-free water and 10 μl PowerTrack SYBR Green Master Mix (Thermo Fisher Scientific). 40 cycles of three-step qPCR were performed, all samples were run in triplicate. All of the primers were synthesized by the Eurofins Genomics Germany GmbH, the detailed sequences of primers are showed in Table 1.

**TABLE 1:** List of qPCR primers sequences.

Gene	Accession no.	Forward	Reverse
NPRA	NM_012613.1	CCTTTCAGGCTGCCAAAAT	ATCCTCCACGGTGAAGTTGA
NPRB	NM_053838.1	TCTATGCCAAGAAGCTGTGG	CCAGGCCTTCCAAGTAGAAA
NPRC	NM_012868.1	TGACACCATTCGGAGAATCA	CATCTCCGTAAGAAGAACTGTTGA
NEP1	NM_012608.2	GGATCTTGTAAGCAGCCTCAGC	AGTTGGCACACCGTCTCCAG
NEP2	NM_001107997.1	AAGGCGGCAGAGACCAGAGAC	CTTGATGGACTGGATGGCGAACTC
AGTR1a	NM_030985.4	GCTTCAACCTCTACGCCAGTGTG	CGAGACTTCATTGGGTGGACGATG
AGTR2	NM_012494.3	TAGTCTCTCTCTTGCCTTGG	CTGACCTTCTTGGATGCTCT
MAS1	NM_012757.2	TGACAGCCATCAGTGTGGAGA	GCATGAAAGTGCCCACAGGA
ACE1	NM_012544.1	GACGGAAGCATCACCAAGGAGAAC	CTAGGCACTGGAGGGCAGAGAC
ACE2	NM_001012006.1	AAGCCACCTTACGAGCCTCCTG	ACAATGCCAACCACTACCGTTCC
BDKRB1	NM_030851.1	CCAAGACAGCAGTCACCATCAA	CAGCAGGTCCCAGTCTTCTAG
BDKRB2	M59967.2	ATCACCATCGCCAATAACTTCGA	CACCACGCGGCACAG
KLK1	NM_012593.1	GGAGAGTTGGAAGGAGGCAAAGAC	TTGGTGTAGATGGCTGGCATGTTG
KLK10	NM_001004100.1	TCCAGAGCGAGCAACTGAGGTC	GTCGTGTTCATCTGAGCGGAGTG
GAPDH	NM_017008	AGACAGCCGCATCTTCTTGT	CTTGCCGTGGGTAGAGTCAT
18S	X00686	TCAACTTTCGATGGTAGTCGCCGT	TCCTTGGATGTGGTAGCCGTTTCT
mtDNA	NC_001665.2	ACACCAAAAGGACGAACCTG	ATGGGGAAGAAGCCCTAGAA
PGC-1α	NM_031347.1	ATGAATGCAGCGGTCTTAGC	AACAATGGCAGGGTTTGTTC

### Immunoblot assay

Total proteins were extracted from CZ by using the Minute™ total protein extraction kit (Invent Biotechnologies). Protein samples were separated on 10% SDS-PAGE and transferred to PVDF membranes (Merck Chemicals GmbH). After incubation in 5% milk (Carl Roth GmbH) in TBST for 1 h at room temperature, membranes were incubated overnight at 4°C with diluted primary antibodies: Anti-NPR-A+NPR-B antibody (ab139188) (1:300), Anti-NEP1 antibody (ab79423) (1:1000), Anti-ACE1 antibody (ab254222) (1:1000), Anti-ACE2 antibody (ab108252) (1:200), Anti-KLK1 antibody (ab131029) (1:1000), Anti-beta Actin antibody (ab115777) (1:1000) and Anti-alpha Tubulin antibody (ab7291) (1:5000). After washing with TBST three times, membranes were incubated with 1:5000 diluted conjugated peroxidase-labeled secondary antibodies Goat Anti-Mouse IgG H&L (HRP) (ab205719) or Goat Anti-Rabbit IgG H&L (HRP) (ab205718) at room temperature for 1 h, followed by washing with TBST three times. The PVDF membrane was reacted with the Pierce™ ECL Western Blotting Substrate (Thermo Fisher Scientific) for 1 min at room temperature. After absorbing the liquid, blots were visualized by using the VWR^®^ Imager CHEMI Premium (VWR International) system, and analyzed by using the Quantity One Software (Bio-Rad Laboratories). All the primary and secondary antibodies were purchased from Abcam. Each experiment for target protein analysis was repeated three times.

### Enzyme-linked immunosorbent assay

Total proteins were extracted from CZ by using the Minute™ total protein extraction kit (Invent Biotechnologies). Quantification of total proteins was achieved by using the Pierce™ BCA™ Protein-Assay (Thermo Fisher Scientific). Measurements of protein concentrations of granulocyte-macrophage colony-stimulating factor (GM-CSF), monocyte chemoattractant protein-1 (MCP-1) and vascular endothelial growth factor (VEGF) were performed by using the Rat GM-CSF ELISA Kit (Assay Genie, RTFI00020), MCP1 (CCL2) Rat ELISA Kit (Abcam, ab100778) and VEGF Rat ELISA Kit (Abcam, ab100787), respectively. Concentrations were measured spectrophotometrically by light absorbance using the Spark multimode microplate reader (Tecan Group AG). All samples were run in triplicate. The final concentration was expressed as pg/μg total protein.

### Analysis of mitochondrial DNA copy number

Two cubic millimeter tissue from LV were homogenized by using the liquid nitrogen grinding method. Genomic DNA was extracted by using the DNeasy Blood & Tissue Kit (Qiagen) according to the manufacturer’s instructions. Quantitative analysis of DNA was performed using the Nanodrop™ Microvolume Spectrophotometer (Thermo Fisher Scientific). DNA was further diluted to a final concentration of 50 ng/μl. The mitochondrial copy number (mtDNA-CN) was expressed relative to a nuclear DNA specific gene proliferator-activated receptor-γ coactivator-1α (PGC-1α). Here, mtDNA-CN was calculated according to the formula: mtDNA-CN = 2 × 2^ΔCt^, ΔCT = CT_*PGC–*1α_ – CT_*mtDNA*_. 60 cycles of a two-step qPCR were performed.

### Cell culture and treatment

Murine heart endothelial cells (MHEC5-T) (Leibniz Institute DSMZ-German Collection of Microorganisms and Cell Cultures GmbH) were grown in RPMI 1640 medium (Thermo Fisher Scientific) containing 10% fetal bovine serum (Sigma-Aldrich Chemie GmbH). Cells were treated with 0.01, 0.1, 1, 10, and 20 μM of the compounds as follows: Ramipril (ACEi) (Sigma-Aldrich Chemie GmbH), Valsartan (ARB) (Sigma-Aldrich Chemie GmbH), Sacubitril calcium salt (NEPi) (Sigma-Aldrich Chemie GmbH), Valsartan and Sacubitril calcium salt (ARNi), R715 [antagonist of BDKRB1 (BDKRB1i)] (Tocris Bioscience) and HOE 140 [antagonist of BDKRB2 (BDKRB2i)] (Enzo Life Sciences GmbH) to determine the optimal concentration.

### Cell proliferation assay

MHEC5-T were cultured in a 96-well plate with 4000 cells per well. After 4 h attachment, cells were cultured in RPMI-1640 medium alone as control or containing 0.01 μM Ramipril (ACEi), 0.01 μM Ramipril and 0.01 μM R715 (ACEi + BDKRB1i), 0.01 μM Ramipril and 0.01 μM HOE 140 (ACEi + BDKRB2i), 0.01 μM Ramipril, 0.01 μM R715 and 0.01 μM HOE 140 (ACEi + BDKRB1i + BDKRB2i); 0.01 μM Sacubitril calcium salt (NEPi), 0.01 μM Sacubitril calcium salt + 0.01 μM R715 (NEPi + BDKRB1i), 0.01 μM Sacubitril calcium salt and 0.01 μM HOE 140 (NEPi + BDKRB2i), 0.01 μM Sacubitril calcium salt, 0.01 μM R715 and 0.01 μM HOE 140 (NEPi + BDKRB1i + BDKRB2i). Cell proliferation was assessed by using the WST-1 Assay Kit (Cell Proliferation) (Abcam) according to the manufacturer’s instructions. Absorbance was spectrophotometrically measured at 450 nm by using the Spark multimode microplate reader (Tecan Group AG), and expressed as optical density (O.D.).

### Statistical analysis

All statistical analyses were performed by using IBM SPSS 26 or Graphpad prism 9. Relative mRNA expression fold change and mtDNA-CN were given as mean ± standard error of the mean (SEM), other parameters were given as mean ± standard deviation (SD). Kolmogorov–Smirnov test was performed to analyze the distribution of quantitative variables. Normally distributed data (ΔMMP, protein expression levels, O.D. values of cytokines concentrations and cell proliferation) were analyzed by one-way analysis of variance (Fisher’s protected least significant difference test), abnormally distributed data (Relative mRNA expression fold change and mtDNA-CN) were analyzed by Kruskal–Wallis test. Comparison of MMP between Day 0 and Day 7 was performed using a paired *t*-test. *P*-values less than 0.05 (≤0.05) were considered to be statistically significant.

## Results

### Angiotensin receptor-neprilysin inhibitors markedly improve coronary collateral perfusion

**FIGURE 3:**
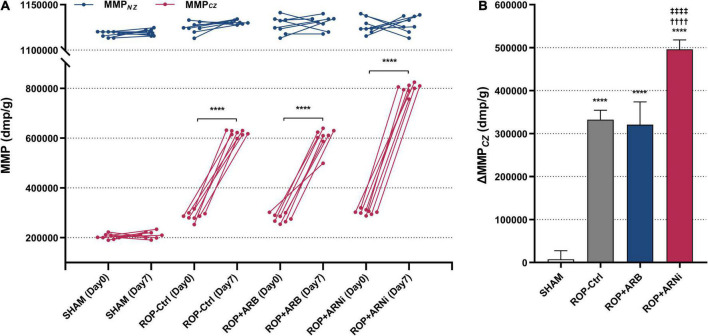
Measurement of myocardial collateral perfusion. MMP, microspheres-based myocardial perfusion; MMP*_*CZ*_*, MMP of CZ; MMP*_*NZ*_*, MMP of NZ; ΔMMP*_*CZ*_*, difference value of MMP*_*CZ*_* at Day 7 and Day 0. **(A)** Microspheres-based myocardial perfusion at Day 0 and Day 7. Blue lines indicate changes of MMP*_*NZ*_* from Day 0 to Day 7, red lines indicate the changes of MMP*_*CZ*_* from Day 0 to Day 7. ^****^P ≤ 0.0001, compared to Day 0. **(B)** Difference value of MMP*_*CZ*_* among groups. ^****^P ≤ 0.0001, compared to SHAM, ^††††^*P* ≤ 0.0001, compared to ROP-Ctrl, ^‡‡‡‡^*P* ≤ 0.0001, compared to ROP+ARB.

First, MMP*_*CZ(D*7)_* was significantly higher than MMP*_*CZ(D*0)_* in all ROP groups, but it was unchanged in the SHAM group (Figure 3A and Table 2). Moreover, ΔMMP*_*CZ*_* in all ROP groups were significantly greater than in the SHAM group. In addition, ΔMMP*_*CZ*_* in the ROP+ARNi group was significantly greater than in the ROP-Ctrl group and ROP+ARB group. Although ΔMMP*_*CZ*_* in the ROP+ARB group was significantly greater than in the SHAM group, it remained unchanged when compared with the ROP-Ctrl group (Figure 3B and Table 2). In contrast, MMP*_*NZ(D*7)_* was unchanged compared with MMP*_*NZ(D*0)_* in all groups (Figure 3A). With regard to ΔMMP*_*NZ*_*, no significant difference was detected between all groups (Table 2).

**TABLE 2:** Measurement of the coronary collateral perfusion.

Group	MMP*_*CZ(D*0)_* (dmp/g)	MMP*_*CZ(D*7)_* (dmp/g)	Δ MMP*_*CZ*_* (dmp/g)	MMP*_*NZ(D*0)_* (dmp/g)	MMP*_*NZ(D*7)_* (dmp/g)	Δ MMP*_*NZ*_* (dmp/g)
SHAM	203626 ± 10391	211218 ± 14218	7591 ± 19957	1117493 ± 3072	1119576 ± 3299	2084 ± 4451
ROP-Ctrl	287118 ± 18279	619780 ± 11321^####^	332663 ± 21728^****^	1124653 ± 6334	1130470 ± 2118	5818 ± 6984
ROP+ARB	279846 ± 17332	600495 ± 44262^####^	320649 ± 53317^****^	1127521 ± 9373	1130063 ± 7646	2541 ± 11744
ROP+ARNi	303161 ± 10094	799348 ± 20406^####^	496186 ± 21723^****††††‡‡‡‡^	1126644 ± 8494	1128854 ± 9321	2210 ± 16523

MMP, microspheres-based myocardial perfusion; MMP_*CZ(D*0)_, MMP of CZ at Day 0; MMP_*CZ(D*7)_, MMP of CZ at Day 7; ΔMMP_*CZ*_, difference value between MMP_*CZ(D*7)_ and MMP_*CZ(D*0)_; MMP_*NZ(D*0)_, MMP of NZ at Day 0; MMP_*NZ(D*7)_, MMP of NZ at Day 7; ΔMMP_*NZ*_, difference value between MMP_*NZ(D*7)_ and MMP_*NZ(D*0)_. ^####^*P* ≤ 0.0001, compared to Day 0, ^****^P ≤ 0.0001, compared to SHAM, ^††††^*P* ≤ 0.0001, compared to ROP-Ctrl, ^‡‡‡‡^*P* ≤ 0.0001, compared to ROP+ARB.

### Angiotensin receptor-neprilysin inhibitors regulate natriuretic peptide system, renin-angiotensin-aldosterone system, and kallikrein-kinin system

**FIGURE 4:**
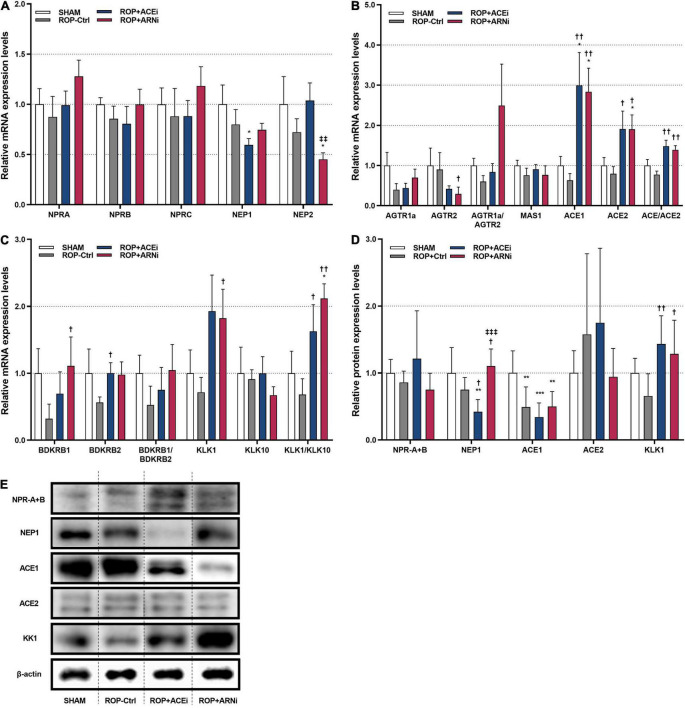
Molecular and biochemical quantification of target molecules of NPS, RAAS, and KKS. **(A)** Relative mRNA expression levels of membrane receptors and key enzymes in NPS. **(B)** Relative mRNA expression levels of membrane receptors and key enzymes in RAAS. **(C)** Relative mRNA expression levels of membrane receptors and key enzymes in KKS. **(D,E)** Relative protein expression levels of target molecules. **(A–C)** Data were given as mean ± standard error of the mean (SEM) and presented here as 2^– Δ^ ^CT^ values and normalized against SHAM. *P ≤ 0.05, **P ≤ 0.01, ***P ≤ 0.001, compared to SHAM. ^†^*P* ≤ 0.05, ^††^*P* ≤ 0.01, compared to ROP-Ctrl. ^‡‡^*P* ≤ 0.01, ^‡‡‡^*P* ≤ 0.001, compared to ROP+ACEi.

mRNA and protein expression levels of the membrane receptors and key enzymes in NPS, RAAS, and KKS were analyzed, respectively (Figure 1). With regard to NPS, results showed that the mRNA expression levels of natriuretic peptide A receptor (NPRA), natriuretic peptide B receptor (NPRB) and natriuretic peptide C receptor (NPRC) in the ROP+ARNi group were highest among all groups, but these results were statistically non-significant. The mRNA expression levels of neprilysin-1 (NEP1) in the ROP+ACEi group (0.60-fold ± 0.06) were significantly lower than in the SHAM group (1.00-fold ± 0.19) (*P* = 0.0372). In addition, the mRNA expression levels of neprilysin-2 (NEP2) in the ROP+ARNi group (0.45-fold ± 0.06) were significantly lower than in the SHAM group (1.00-fold ± 0.28) (*P* = 0.0275) and the ROP+ACEi group (1.04-fold ± 0.18) (*P* = 0.0052) (Figure 4A). Moreover, results from immunoblot assay indicated that the protein expression levels of NPRA + NPRB were unchanged between all groups. However, the protein expression levels of NEP1 in the ROP+ACEi group (0.42-fold ± 0.18) were significantly lower than in the SHAM group (1.00-fold ± 0.38) (*P* = 0.0023), ROP-Ctrl group (0.75-fold ± 0.18) (*P* = 0.0489), and ROP+ARNi group (1.11-fold ± 0.25) (*P* = 0.0004), respectively (Figures 4D, E).

With regard to RAAS, results showed that the mRNA expression levels of angiotensin II receptor type 1a (AGTR1a) in either the ROP+ACEi group or the ROP+ARNi group were higher than in the ROP-Ctrl group, but no significant differences were detected. In contrast, the mRNA expression levels of angiotensin II receptor type 2 (AGTR2) in the ROP+ARNi group (0.29-fold ± 0.17) were significantly lower than in the ROP-Ctrl group (0.90-fold ± 0.42) (*P* = 0.0412). In consequence, the relative mRNA expression ratios of AGTR1a/AGTR2 in the ROP+ARNi group were highest among all groups. In addition, the mRNA expression levels of angiotensin converting enzyme 1 (ACE1) in the ROP+ACEi group (3.00-fold ± 0.81) were significantly higher than in the ROP-Ctrl group (0.63-fold ± 0.17) (*P* = 0.0037) and in the SHAM group (1.00-fold ± 0.22) (*P* = 0.0275). Moreover, ACE1 mRNA expression levels were also significantly higher in the ROP+ARNi group (2.84-fold ± 0.59) compared with the ROP-Ctrl group (*P* = 0.0033) and the SHAM group (*P* = 0.0247). Similarly, the mRNA expression levels of angiotensin converting enzyme 2 (ACE2) in the ROP+ACEi group (1.91-fold ± 0.44) were significantly higher than in the ROP-Ctrl (0.79-fold ± 0.18) (*P* = 0.0373). Besides, ACE2 mRNA expression levels were significantly higher in the ROP+ARNi group (1.91-fold ± 0.36) compared with the SHAM group (1.00-fold ± 0.20) (*P* = 0.0373) and the ROP-Ctrl group (*P* = 0.0143). As a consequence, the relative mRNA expression ratios of ACE1/ACE2 in either the ROP+ACEi group (1.48-fold ± 0.15) (*P* = 0.0017) or the ROP+ARNi group (1.39-fold ± 0.11) (*P* = 0.0023) were significantly higher than in the ROP-Ctrl group (0.77-fold ± 0.09) (Figure 4B). Moreover, the protein expression levels of ACE1 in the SHAM group (1.00-fold ± 0.33) were significantly higher than in the ROP-Ctrl group (0.49-fold ± 0.30) (*P* = 0.0058), ROP+ACEi group (0.34-fold ± 0.21) (*P* = 0.0008), and the ROP+ARNi group (0.50-fold ± 0.22) (*P* = 0.0086), respectively (Figures 4D, E).

Finally, with regard to KKS, the mRNA expression levels of BDKRB1 in the ROP+ARNi group (1.11-fold ± 0.43) were significantly higher than in the ROP-Ctrl group (0.32-fold ± 0.22) (*P* = 0.0367). BDKRB1 mRNA expression levels were also higher in the ROP+ACEi group compared with the ROP-Ctrl group, but the results were without any statistical significance. In addition, the mRNA expression levels of BDKRB2 in the ROP+ACEi group (1.00-fold ± 0.15) were significantly higher than in the ROP-Ctrl group (0.56-fold ± 0.08) (*P* = 0.0453). BDKRB2 mRNA expression levels were slightly higher in the ROP+ARNi group compared with the ROP-Ctrl group. In addition, the relative mRNA expression ratios of BDKRB1/BDKRB2 in either the ROP+ACEi group or the ROP+ARNi group were higher than in the ROP-Ctrl group, but no statistical significance was reached. Moreover, the mRNA expression levels of kallikrein 1 (KLK1) in the ROP+ARNi group (1.82-fold ± 0.43) were significantly higher than in the ROP-Ctrl group (0.72-fold ± 0.22) (*P* = 0.0500). In addition, the mRNA expression levels of kallikrein 10 (KLK10) were unchanged between all groups. As a consequence, the relative mRNA expression ratios of KLK1/KLK10 in the ROP+ACEi group (1.63-fold ± 0.40) were significantly higher than in the ROP-Ctrl group (0.68-fold ± 0.24) (*P* = 0.0275). The KLK1/KLK10 mRNA expression ratios were also significantly higher in the ROP+ARNi group (2.12-fold ± 0.22) compared with the SHAM group (1.00-fold ± 0.33) (*P* = 0.0412) and the ROP-Ctrl group (*P* = 0.0019) (Figure 4C). Furthermore, the protein expression levels of KLK1 in both the ROP+ACEi group (1.43-fold ± 0.42) (*P* = 0.0043) and the ROP+ARNi group (1.29-fold ± 0.50) (*P* = 0.0165) were higher than in the ROP-Ctrl group (0.66-fold ± 0.33) (Figures 4D, E).

### Angiotensin receptor-neprilysin inhibitors increase pro-arteriogenic cytokines concentrations

**FIGURE 5:**
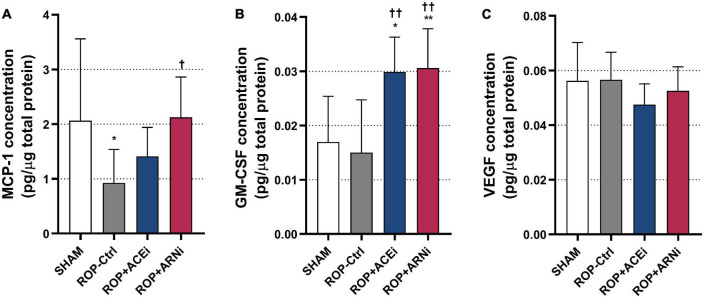
Analysis of pro-arteriogenic cytokines. **(A–C)** Protein concentrations of pro-arteriogenic cytokines. *P ≤ 0.05, **P ≤ 0.01, compared to SHAM. ^†^*P* ≤ 0.05, ^††^*P* ≤ 0.01, compared to ROP-Ctrl.

MCP-1 concentration in the ROP+ARNi group (2.13 ± 0.74 pg/μg total protein) was significantly higher than in the ROP-Ctrl group (0.92 ± 0.61 pg/μg total protein) (*P* = 0.0359). Moreover, MCP-1 concentration in the ROP-Ctrl group was markedly lower than in the SHAM group (2.06 ± 1.50 pg/μg total protein) (*P* = 0.0457) (Figure 5A). In addition, GM-CSF concentration in the ROP+ACEi group (0.0299 ± 0.0064 pg/μg total protein) was significantly higher than in the SHAM group (0.0170 ± 0.0084 pg/μg total protein) (*P* = 0.0116) and the ROP-Ctrl group (0.0150 ± 0.0097 pg/μg total protein) (*P* = 0.0045). GM-CSF concentration was significantly higher in the ROP+ARNi (0.0306 ± 0.0072 pg/μg total protein) compared with the SHAM group (*P* = 0.0081) and ROP-Ctrl (*P* = 0.0031) (Figure 5B). Regarding VEGF concentration, results showed that there was no statistical significance between all groups (Figure 5C).

### Angiotensin receptor-neprilysin inhibitors hardly affect mitochondrial genome synthesis

mtDNA-CN in either the ROP+ACEi group (0.73-fold ± 0.07) or the ROP+ARNi group (0.77-fold ± 0.12) was slightly lower than in either the SHAM group (1.00-fold ± 0.10) or the ROP-Ctrl (0.98-fold ± 0.11), however, these results were not statistically significant.

### Neprilysin inhibitors exert pro-arteriogenic effects through the bradykinin receptor signaling pathway

**FIGURE 6:**
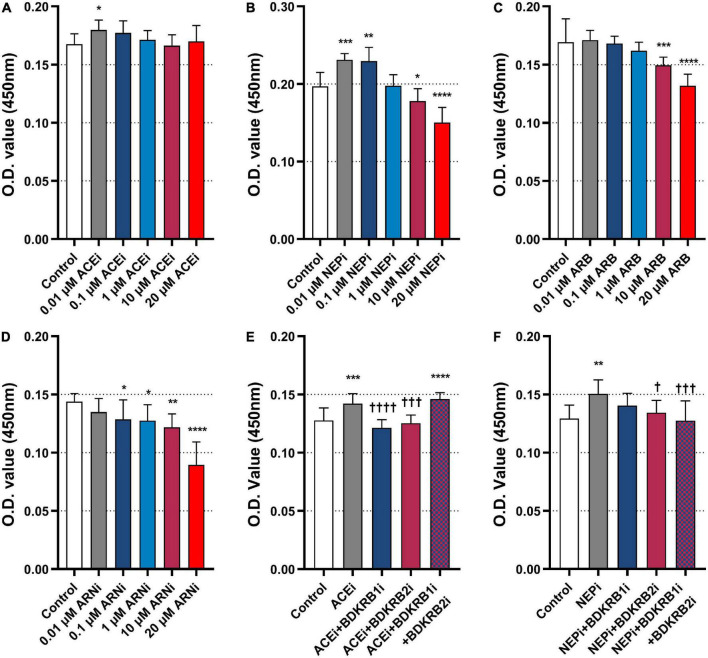
Effects of ACEi, NEPi, ARB, ARNi, and antagonists of bradykinin receptors on MHEC5-T proliferation. **(A–D)** Effects of ACEi, NEPi, ARB, ARNi on MHEC5-T proliferation at different concentrations, *P ≤ 0.05, **P ≤ 0.01, ***P ≤ 0.001, ****P ≤ 0.0001, compared to control. **(E)** Effects of ACEi and antagonists of bradykinin receptors on MHEC5-T proliferation. ***P ≤ 0.001, ****P ≤ 0.0001, compared to control. ^†††^*P* ≤ 0.001, ^††††^*P* ≤ 0.0001, compared to ACEi. **(F)** Effects of NEPi and antagonists of bradykinin receptors on MHEC5-T proliferation. **P ≤ 0.01, compared to control. ^†^*P* ≤ 0.05, ^†††^*P* ≤ 0.001, compared to NEPi.

*In vitro* experiments were performed to investigate the roles of ACEis, ARBs, NEPis, and ARNis on MHEC5-T proliferation. The results showed that 0.01 μM ACEi (0.1797 ± 0.0086) significantly promoted cell proliferation compared with the control (0.1677 ± 0.0088) (*P* = 0.0196) (Figure 6A). Similarly, both 0.01 μM NEPi (0.2311 ± 0.0080) (*P* = 0.0009) and 0.1 μM NEPi (0.2294 ± 0.0177) (*P* = 0.0014) significantly promoted cell proliferation compared with the control (0.1969 ± 0.0180) (Figure 6B). In contrast, both 10 μM ARB (0.1495 ± 0.0069) (*P* = 0.0008) and 20 μM ARB (0.1318 ± 0.0101) (*P* < 0.0001) significantly inhibited cell proliferation compared with the control (0.1693 ± 0.0202) (Figure 6C). Moreover, ARNi (NEPi + ARB) significantly inhibited cell proliferation in a concentration-dependent manner from 0.01 to 20 μM (Figure 6D).

Then, we functionally validated whether the pro-arteriogenic effects of ACEis and ARNis were mediated through the bradykinin signaling pathway. The results showed that ACEi (0.1421 ± 0.0086) significantly promoted cell proliferation compared with control (0.1276 ± 0.0109) (*P* = 0.0010). In contrast, ACEi in combination with BDKRB1i (0.1213 ± 0.0070) (*P* < 0.0001) or BDKRB2i (0.1254 ± 0.0069) (*P* = 0.0002) significantly inhibited cell proliferation compared with ACEi alone. However, ACEi in combination with BDKRB1i and BDKRB2i (0.1460 ± 0.0055) showed a markedly increased cell proliferation when compared with control (*P* = 0.0001) (Figure 6E). Furthermore, NEPi (0.1505 ± 0.0119) significantly promoted cell proliferation compared with control (0.1294 ± 0.0115) (*P* = 0.0018). In contrast, NEPi in combination with BDKRB2i (0.1344 ± 0.0105) (*P* = 0.0141), or NEPi in combination with BDKRB1i and BDKRB2i (0.1276 ± 0.0169) (*P* = 0.0008) significantly inhibited cell proliferation compared with NEPi alone (Figure 6F).

## Discussion

In case of coronary occlusion, collateral growth is the most efficient compensatory mechanism to adequately supply blood to ischemic myocardium. This study demonstrated for the first time that ARNis significantly improve coronary collateral perfusion *in vivo*. Furthermore, we demonstrated that NEPis exert pro-arteriogenic efforts *via* the bradykinin receptor signaling pathway *in vitro*.

In our current research, we validated again that ROP is an ideal approach to stimulate coronary collateral growth. Most importantly, administration of 7-day ROP+ARNi significantly increased coronary collateral perfusion compared with ROP alone. In contrast, ROP+ARB had no effect on coronary collateral perfusion. Thus, we confirmed that the ARNi induced improvement in coronary collateral perfusion was due to the NEPi (Sacubitril), rather than the ARB (Valsartan). This result is consistent with our most recent study, in which a beneficial effect of an ARB (Candesartan) on cerebral collateral blood flow was not observed (11).

Neprilysin degrades both BK and NPs, and there is overwhelming evidence that both BK and NPs are the most potent endogenous vasodilators modulating coronary blood flow (CBF). It has been well documented that exogenous BK increased CBF in a dose-dependent manner (16), while NPs enhanced coronary artery dilation and increased coronary flow velocity (17, 18). The results presented here were obtained in an established rat model of coronary arteriogenesis. Hence, the improvement in myocardial perfusion was clearly the result of chronic remodeling of the coronary collateral arteries rather than a transient regulation of vascular tone. BK and NPs exert their biological functions by binding bradykinin receptors and natriuretic peptide receptors, respectively. Indeed, increasing evidence suggests that both bradykinin receptors and natriuretic peptide receptors play critical role in collateral artery development (7, 19, 20).

Therefore, in the second part, by analyzing the relevant membrane receptors and key enzymes in NPS, RAAS and KKS, we aimed to understand the underlying molecular mechanism and cross-talk in these three hormonal systems under the administration of ACEis or ARNis. First, NPS is the most prevalent system regulated by ARNis. Hence, we analyzed three major membrane receptors (NPRA, NPRB, NPRC) in NPS, and the results showed that their mRNA expression levels were highest in the ROP+ARNi group among all groups, but a statistical significance was not confirmed. NEP1 is one of the key degradative enzymes in NPS. Our results showed that ACEis, but not ARNis, significantly inhibited NEP1 expression at both mRNA and protein expression levels. Yet, ARNis significantly inhibited the mRNA expression levels of NEP2. Currently, there are no specific studies that clarify the effect of ACEis on the NEP family. However, Pare et al. analyzed the candidate genes associated with ACEi-induced angioedema in a genome-wide study, and the results showed that NEP variants were likely involved in ACEi-induced immunoregulation (21), which may explain the putative ACEi-modulated downregulation of NEP1 mRNA expression observed in our study. Regarding NEP2, Bland et al. demonstrated a high expression of NEP2 in the soluble melanogaster embryo fraction (22). Our previous *in vivo* study also demonstrated that embryonic stage is the most critical phase for arterial identity (23). To date, however, little is known about NEP2 in the context of vascular adaptations.

Second, with respect to RAAS, the main membrane receptors are AGTR1a (AGTR1 consists of AGTR1a and AGTR1b, the first subunit is strongly expressed in the heart) and AGTR2 (24). Our results showed that ARNis significantly downregulated AGTR2 mRNA expression and led to an upregulation of the AGTR1a/AGTR2 ratio. In principle both Ang I and Ang II are cleaved by NEP, so inhibition of NEP increases their concentrations in the circulation system. Indeed, it was reported that NEPis increased blood pressure in normotensive subjects (25), which was identified as an Ang II-dependent effect (26). Although both AGTR1 and AGTR2 have the similar binding affinity for Ang II, they exert opposite biological functions in cardiovascular homeostasis (27). However, an imbalance of the AGTR1/AGTR2 ratio should be theoretically reversed when the ARB (Valsartan) is combined with the NEPi (Sacubitril). In this regard, numerous studies have shown that the cardiovascular protective function of ARBs was partly due to the enhancement of the biological effect of AGTR2 (28). In addition, recent preclinical studies indeed demonstrated that ARNis downregulated AGTR1 expression but upregulated AGTR2 expression at the transcriptional level (29, 30), which are in contrast to our finding. Here, the underlying mechanism is still not clear.

With regard to the key enzymes in RAAS, our results showed that the mRNA expression levels of both ACE1 and ACE2 were upregulated under the administration of either ACEis or ARNis, and a greatly increased ACE1/ACE2 ratio was observed. Indeed, early in the development of ACEis, it was reported that administration of ACEis upregulated ACE mRNA (31, 32), which was considered as a negative feedback effect of inhibited ACE activity and decreased Ang II levels (33). However, studies subsequently reported that ACEis decreased ACE1 mRNA expression but increased ACE2 (34, 35). It is now generally accepted that ACE2 converts Ang II to Ang-(1-7), which is a potent vasodepressor that counteracts the vasopressor Ang II. Therefore, ACEis exert an important cardiovascular protective function by regulating the imbalance of the ACE1/ACE2 ratio (36). In contrast, Emilsson et al. recently reported that ACEis even increased serum protein levels of ACE1, whereas ACE2 levels remained unchanged (37). Still, the roles of ACEis and ARNis on ACEs expression in this context remain controversial.

Third, to verify our hypothesis that ARNis functionally activate KKS like ACEis, we analyzed the mRNA expression of bradykinin receptors and two members of the tissue kallikrein family. Our results showed that the gene expression of bradykinin receptors was modulated by both ARNis and ACEis. Notably, an upregulated mRNA expression of BDKRB1 was observed in the ROP+ARNi group, while an upregulated mRNA expression of BDKRB2 was observed in the ROP+ACEi group, respectively.

The mechanism of ACEis on bradykinin receptor activation has been intensively investigated in numerous studies. In summary, as agonists, ACEis directly active BDKRB1 by binding the zinc finger motif of the second extracellular loop. As allosteric enhancers, ACEis indirectly resensitize BDKRB2 by altering the conformation of ACE domains of the ACE-BDKRB2 receptor heterodimer. Here, the possible molecular mechanisms of NEPis on bradykinin receptor activation seem conceivable. First, inhibition of NEP leads to increased BK concentration levels, which thereby stabilizing and activing BDKRB1 and BDKRB2. Second, it is speculated that NEP may also associate with BDKRB2 to form a “NEP-BDKRB2 heterodimer,” thereby enhancing peptide ligand binding and activating BDKRB2 (38). Intriguingly, it has been reported that NEP was much more responsible for kininase function (68 ± 2%) than ACE (9 ± 0.4%) in the murine kidney (39). If NEP has a stronger effect on BK degradation than ACE in the heart, we speculate that NEPis may be more efficient than ACEis to stimulate coronary arteriogenesis therapeutically in ischemic cardiovascular disease, and likely through the bradykinin receptor signaling pathway.

Previous research demonstrated that NEPis could augment the beneficial effect of KKS by particularly stimulating BDKRB2. Ura et al. reported that NEPi-induced increase in renal kinin levels were blocked by the BDKRB2 antagonist (HOE140) (40). In addition, Deddish et al. demonstrated that NEPis resensitized BDKRB2 in human pulmonary fibroblasts (38). However, in our current study, only an upregulated BDKRB1 mRNA expression was detected under the administration of ARNi, suggesting that BDKRB1 plays a greater role than BDKRB2 in ARNi-induced arteriogenesis. Indeed, we previously observed a large reduction in peripheral arteriogenesis in BDKRB1 knock out mice, and a minor reduction in BDKRB2 knock out mice (7). Therefore, BDKRB1 can be regarded as a novel pro-arteriogenic therapeutic target in GPCR drug discovery.

Furthermore, our work showed that both ACEis and ARNis regulated gene expression of the kallikreins, KLK1 and KLK10. KLK1 converts low-molecular-weight kininogen (LMWK) to KD. KD is the ligand of BDKRB2, which is known to exert numerous biological processes implicated in vascular growth (41). In contrast, several studies concluded that KLK10 is a tumor suppressor gene, which is a major modulator of inhibition of vascular cell proliferation and migration (42, 43). Theoretically, downregulation of KLK10 mRNA levels could be associated with vascular cell proliferation and migration, which are the prerequisites of arteriogenesis. Therefore, we considered the mRNA expression ratio of KLK1/KLK10 as a pro-arteriogenic indicator. Here, our results showed for the first time that both ACEis and ARNis significantly upregulated this pro-arteriogenic ratio. In addition, our results also showed that both ACEis and ARNis upregulated KLK1 at the translational level.

Many research has been made regarding the expression ratio of AGTR1/AGTR2, ACE1/ACE2, and BDKRB1/BDKRB2 (44–46). Upregulation or imbalance of these ratios was considered a hallmark of cardiac decompensation and arterial inflammation. Most investigators attributed the pharmacological benefits of ACEis or ARBs to rebalancing of these ratios (34). However, arteriogenesis is a process in which immune activation and inflammatory activation play crucial roles. In particular, leukocyte extravasation is triggered after arterial occlusion. Subsequently, monocytes adhere and transmigrate across vascular endothelium, and differentiate into macrophages, which release numerous cytokines (e.g., GM-CSF, MCP-1, and VEGF). These pro-arteriogenic cytokines significantly promote ECs proliferation *via* the paracrine signaling processes (47). In summary, at the mechanistic level, there are both beneficial and adverse effects of ACEis and ARNis therapy, but ultimately the beneficial effects predominate, leading to improved collateral formation.

Because paracrine factors play a critical role in arteriogenesis, we analyzed whether administration of ACEis or ARNis would result in therapeutic modulation of three main pro-inflammatory cytokines at the protein level. Our results suggested that the pro-arteriogenic cytokines were strongly induced during therapeutic modulation of arteriogenesis. Here, we demonstrated that ARNis, but not ACEis, significantly increased MCP-1 concentration, and that both ACEis and ARNis increased GM-CSF concentration. It has been reported that MCP-1 plays an important role in monocyte/macrophage activation, and that GM-CSF stimulates the release of pluripotent monocyte cells from the bone marrow into the collateral circulation (48–50). The therapeutic and pro-arteriogenic roles of GM-CSF and granulocyte colony-stimulating factor (G-CSF) have been demonstrated in a variety of animal models of coronary, cerebral, and peripheral arteriogenesis, respectively (13, 51, 52). Yet, a modulation of VEGF at the protein level by ACEis or ARNis was not confirmed in our current study.

Vascular regeneration can be regarded as a plastic process, in which physiological and pathophysiological processes work against each other, ultimately the former gains the upper hand (53). Mitochondrial dysfunction is a hallmark of age-related cardiovascular disease. In fact, the heart is a “muscle pump” that constantly has an extraordinarily high demand for adenosine triphosphate, which is why it has a high density of mitochondria (54). Sabbah et al. demonstrated that ARNis could ameliorate left ventricular mitochondrial dysfunction (55). However, there was no study demonstrating the role of ARNis in mitochondrial biogenesis, whereas previous research has shown that administration of ACEis increased mtDNA-CN (56, 57). Yet, a beneficial effect of ARNis or ACEis on myocardial mitochondrial biogenesis was not confirmed in our current study.

To verify our hypothesis that ARNis exert pro-arteriogenic effects *via* the bradykinin receptor signaling pathway, additional *in vitro* experiments were performed in this study. Because it was confirmed here that NEPis, rather than ARBs, promoted endothelial proliferation, the NEPi (Sacubitril) was analyzed for the possible stimulation of bradykinin receptors. Hence, in subsequent *in vitro* experiments, we verified our hypothesis that NEPis, like ACEis, exert their biological function on ECs through the bradykinin receptor signaling pathway. Here, we have shown for the first time that NEPis significantly promoted MHEC5-T proliferation, which can be abrogated by antagonists of bradykinin receptors. Thus, it can be demonstrated that NEPis exert pro-arteriogenic effects *via* the bradykinin receptor signaling pathway. In particular, since a stronger inhibition of cell proliferation was observed in the NEPi + BDKRB2i treatment group compared with the NEPi + BDKRB1i treatment group, BDKRB2 plays a greater role than BDKRB1 in NEPi-induced endothelial proliferation.

Vascular proliferation and migration play crucial roles in various contexts of arterial remodeling. The endothelium is a thin monocellular layer that lines the inner surface of the heart and blood vessels. As a receptor-effector, the endothelium has the property to respond to physical or chemical stimuli. It maintains vasomotor balance and vascular homeostasis by producing agonistic and antagonistic substances (58). Conversely, endothelial dysfunction is characterized by an imbalanced vasodilation and vasoconstriction (59). It has been demonstrated that endothelial dysfunction precedes atherosclerosis (60). Because atherosclerotic lesions result in the migration of vascular smooth muscle cells from the media to intima (61, 62), and endothelial integrity is maintained by replacement of damaged ECs (63), atherosclerosis is characterized by pathologic intimal thickening. In contrast, with regard to arteriogenesis, collateral arterioles undergo active outward remodeling, which is associated with wall thickening and lumen enlargement. Here, ECs proliferation and migration are essential for collateral artery formation. It is speculated that novel medications for cardiovascular disease may shift the process of pathologic atherosclerosis toward physiological arteriogenesis (64). Recent research has shown that arterial network expansion complemented collateral arterial development to recover from an ischemic insult, in which endothelial function plays an important role in arterial flow recovery (65, 66).

From bench to bedside, ARNis were used only for HF patients with reduced ejection fraction (HFrEF) at the very beginning after it appeared. On the positive side, the indications of ARNis were expanded for both HF with preserved ejection fraction (HFpEF) (approved by the U.S. Food and Drug Administration) (67) and hypertension (approved by the China Food and Drug Administration) (68) in 2021. In addition, results of the PARADISE-MI trial (*n* = 5661) showed that administration of ARNis reduced the composite outcome by 10% compared with ACEis, and provided additional clinical benefits in patients with acute MI (69). Meanwhile, results from another multi-center randomized control clinical trial conducted in China (*n* = 7556) showed that ARNis were superior to ACEis in reducing major adverse cardiovascular events after MI (70). Considering that our current study suggests that ARNis significantly facilitate coronary collaterals development, which is the most effective mechanism for maintaining stable blood perfusion after arterial stenosis or occlusion, ARNis may improve the prognosis of patients post-MI HF (tertiary prevention), or even reduce the incidence of new-onset MI (secondary prevention). More research is still needed to provide a rationale for the clinical efficacy and safety of ARNi in MI.

## Conclusion

In summary, the results presented here indicate that (1) ARNis improve coronary collateral perfusion by stimulating arteriogenesis therapeutically. (2) NEPis promote endothelial proliferation *via* the bradykinin receptor signaling pathway.

## Limitations

First, since our primary finding clearly showed that ARNis significantly increased coronary arteriogenesis, it would be supportive to demonstrate the increase in vessel lumen of collateral arteries angiographically. We have already successfully verified the morphological features by visualizing angioarchitecture in a rat model of cerebral arteriogenesis (7, 47). However, anatomically, the situation of rat coronary collaterals is more complicated. Second, it would be more promising to evaluate the role of ARNis on coronary collateral perfusion by using the bradykinin receptor knock out mouse model. However, it is too challenging to perform microsurgery and set up ROP system in the heart of a mouse. Finally, because antibodies against some targets are not available, and the specificity of some antibodies has been controversial, the analysis of these targets at the translational level could only be partial.

## Data availability statement

The original contributions presented in this study are included in this article/Supplementary material, further inquiries can be directed to the corresponding authors.

## Ethics statement

The animal study was reviewed and approved by the State Office for Health and Social Affairs (Landesamt für Gesundheit und Soziales), Berlin, Germany.

## Author contributions

All authors discussed and participated to draft, revise, and submit the manuscript.
